# Effect of Integrative Neuromuscular Training for Injury Prevention and Sports Performance of Female Badminton Players

**DOI:** 10.1155/2021/5555853

**Published:** 2021-04-23

**Authors:** Wei Zhao, Changquan Wang, Yan Bi, Lianxu Chen

**Affiliations:** ^1^College of Physical Education and Sports, Beijing Normal University, Beijing, China; ^2^School of Physical Education, Northwest Normal University, Lanzhou, China; ^3^Institute of Sports Medicine, Beijing Tsinghua Changgung Hospital, Beijing, China

## Abstract

**Objectives:**

Investigate the effects of integrated neuromuscular training (INT) on injury prevention and the performance of professional female badminton athletes by comparing their preintervention and postintervention tests. The study hypothesized that integrated neuromuscular training can improve the asymmetry and improve the sport performance of female badminton players.

**Methods:**

According to pretest value based on functional movement screening, 38 participants were divided into a high-risk group (HG) and a low-risk group (LG) with 22 and 16 people in each group. Two groups of athletes took part in an 8-week INT program consisting of four 90-min sessions each week. The asymmetries in movement, physical fitness, and special abilities were tested before and after the intervention. Independent sample *t*-test was used for the statistical analysis.

**Results:**

This research found indicated that 8 weeks of INT influenced FMS scores in both groups (HG and LG). The change of inline lunge (ES_*H*_ = 0.42, ES_*L*_ = 0.21) and trunk stability push-up (ES_*H*_ = −0.58, ES_*L*_ = −0.20) showed significant differences (*P* < 0.05), and the change of the FMS scores (ES_*H*_ = 0.81, ES_*L*_ = 0.65), deep squat (ES_*H*_ = 0.6, ES_*L*_ = 0.3), and rotation stability (ES_*H*_ = −0.65, ES_*L*_ = −0.72) showed very significant differences (*P* < 0.01). Compared to the pretest, most of the physical fitness parameters improved significantly in the HG and LG groups except strength index, and special abilities of the HG and LG group women badminton athletes showed a substantial increase.

**Conclusion:**

Integrated neuromuscular training can effectively improve the asymmetry of female badminton athletes' limbs, prevent sports injury, and improve the athlete's performance ability. However, athletes in different risk groups have certain differences in the degree of improvement in their motor skills.

## 1. Introduction

Integrated neuromuscular training (INT) is a combination of functional movement training and specific strength, balance, speed, sensitivity, and isometric training, which aims to evaluate and prevent sports injury and improve sports performance [1, 2]. The separate components of integrative neuromuscular training have been evaluated and recommendations have been made to improve performance in sports activities. The INT approach has been shown to develop an athletes' sense of proprioception. When not implemented, there is a risk of the training of athlete. Due to inflexibility, uncoordinated movement, and limb differences, they have been associated with an increased risk of sports injury (REF). Increase risk of injury can impact skill development and longevity [3, 4]. Studies have shown that INT can effectively prevent and improve recovery from sports injury, and an intervention focusing on integrated neuromuscular training can improve the body's muscle ligament and joint structure to enhance the comprehensive performance ability of the body [5].

Neuromuscular control defects are mainly manifested as decreases in muscle strength, explosive power, or abnormal activation patterns [6]. Participating in sports training is biased towards conventional sports training methods, which focus on the practice of a single muscle group, single joint, physical fitness, and technical tactics. As we all know, ligaments and muscles have a synergistic effect on maintaining the stability of the knee joint [7]. Recent data shows that a complex system sensorimotor synergy may exist around the anatomy, the structure of the knee, including ligaments, antagonists muscle pairs (flexors and extensors), bone and sensation ligaments, joint capsules, and related muscles [8].

The main mechanism of how INT reduces the risk of sports injury and improves sports performance is that the human motor sensory system is stimulated by INT, and complex multisensory information is integrated into the central nervous system to achieve motor control [9]. The stability of the joints mainly depends on the ability of the surrounding muscles to be properly activated at any given moment. A highly dynamic process that includes coordinated interaction between all muscles that support the joint. Studies have emphasized that neuromuscular training can be used as a means to manage motion control, improve the motor and sensory systems, improve the dynamic stability of the joints, and reduce the risk of injuries [9]. Fernandez [10] discussed the effect of 5-week neuromuscular training on different short-distance sprint speeds and special sensitivity tests of young tennis players. The results found that the neuromuscular training arrangement in training before the tennis training is better than the nerves after the tennis training muscle training group. Does neuromuscular training have the same effect in different genders. Trajković [11] used 8 weeks to test how the neuromuscular training may influence the athletic ability and physical performance of female volleyball players aged 10-12. The results found that neuromuscular training during training promotes the athletic performance and physical performance of female volleyball players.

Noncontact anterior cruciate ligament injuries often occur in badminton. The main reasons for knee joint injuries are change of direction or cutting action combined with deceleration, jumping to the ground when close to full extension, and pivoting and standing firm when the knee is close to full extension [2]. Badminton is the sport with the highest rate of acute injury among the sports, accounting for 1-5 percent of total sports injuries [12]. The technical characteristics of the unilateral handhold also affect the imbalanced development of the arms and torso, which is mainly manifested as an imbalance of muscle strength and an asymmetry in the quality of the completion of actions. Besides, any imbalance increases the risk of injuries. Kiesel et al. [13] suggest that fundamental movement patterns and pattern asymmetry are identifiable risk factors for time-loss injury during the preseason in professional football players. Differences in the morphological profiles of young tennis players and how these values can affect coordinative abilities have been identified among chronological age categories [14]. Goh et al. [15] and Phomsoupha and Laffaye [16] researched causes of injuries occurring at different positions of the shuttlecock and found that incorrect technical movement can lead to injury to the upper limbs and shoulder joint, extremities and trunk kinematic chain, wasted energy transfer, and increased stability of the power loss, increasing the risk of damage. The damage occurs at a rate of 2.9 injuries for each player per 1000 hours playing badminton [17]. Balance is a major factor to prevent injury [18]. Therefore, the development of lower limb asymmetries should be expected and taken into consideration in any prevention strategy in badminton.

Exercise has become a very important tool to reinjury in the practice of rehabilitation behavior. At present, there have been studies using functional movement screening (FMS) to identify risk problems in sports performance. Shojaedin et al. [19] researched that an approximately 4.7 times greater chance of suffering a lower extremity injury during a regular competitive season if they score less than 17 on the FMS. Reaching the same conclusion, Chorba et al. [20] study showed that a significant correlation was found between low-scoring athletes and injury (*P* = 0.0214, *r* = 0.76). The FMS test can predict the risk of injury for athletes without a previous record of sports injuries.

At the same time, other studies have shown that neuromuscular and joint injury risk factors can be improved through integrated neuromuscular training, and there is evidence to support the implementation of INT methods to prevent and treat athletes returning to the field after some injuries. The level of basic motor skills lays the foundation for complex body movement and sports training [17]. On the one hand, screening via the FMS can help identify either increased or decreased risk of injury. On the other hand, athletes with excellent athletic performance in competition may perform poorly on the FMS test, reflecting the fact that these athletes have produced more compensatory movements and energy consumption during training and competition and have not effectively mastered the basics. FMS test results are useful in identifying basic movements.

Although INT is very important, a paucity of data exists regarding the relationship between INT and young female badminton players' functional movements and athletic performance. Therefore, the purpose of this study was to examine the effect of INT on the quality of functional movements and performance of young female badminton players. As far as the author knows, there are few studies on the special training of INT for Female badminton. Therefore, people know little about the effects of INT interventions in badminton.

The hypothesis of this research was to integrated neuromuscular training significantly reduces the asymmetry of basic technical movements of badminton players and improves their movement competencies based on FMS.

## 2. Methods

### 2.1. Participant

Thirty-eight youth female badminton players (M ± *SD*, age: 17 ± 1.1 years; height: 1.70 ± 6.5 m; body mass: 58 ± 4.2 kg) were selected from the TJ Provincial Badminton Team. Players had more than 9 years of professional sports training, had no major injuries within the last two months, maintained a regular diet and schedule of work and rest, did not take any form of nutritional supplements or drugs, and were informed about the purpose and process of the experiment. Each participant read and signed an informed consent from testing approved to ensure the smooth progress of the experiment. All procedures are in accordance with the Helsinki's statement.

### 2.2. Integrated Neuromuscular Training Testing Procedures

Before the start of the experiment, the subjects were tested by functional movement screen (FMS), and they were divided into a high-risk group (HG) and a low-risk group (LG) according to the FMS test results (Table 1). Data related to the participants' characteristics including, age, body mass, height, BMI, and training years in addition to the research variables were analyzed using the SPSS software version 21 (Table 2).

Taking into account, the high incidence of sports injuries in badminton, screening via the FMS can help identify either increased or decreased risk of injury. Perform functional movement screen on candidate test subjects before the experiment. Functional movement screen is used as one of the experimental test indicators. After the FMS test, 38 female badminton players were selected to participate in this experimental study.The pretest was performed 1 week before the training intervention, and the posttest was performed 1 week after the intervention. The experimental group took part in an 8-week INT program consisting of four 90-min sessions each week (Table 3). Players participating in the intervention group needed to maintain their current training regime.

The INT design used in this study combined the results of previous studies [2, 9, 21–24] and solicits specific opinions and suggestions from coaches and experts. Its content includes balance ability, coordination ability, plyometric training, speed and agility, and core stability training modules. Every Monday, Tuesday, Thursday, and Friday morning, INT was performed for 90 min after warm-up activities of approximately 15 minutes each. The different training plates, training contents and training loads are shown in Tables 3 and 4. The rest time of each group was 1-2 min. Three minutes of rest were scheduled between the plates, and each training plate includes 5-7 exercises. The sensitivity training, plyometric training, and resistance exercises had sufficient intervals between the groups to ensure the quality of each group. In addition, on the basis of reducing the risk of athletes' injury, it pays attention to the stability and flexibility of the joints and the needs of specific physical fitness, as well as functional exercises on the nongrip side to improve the athlete's balance ability.

### 2.3. Assessments

#### 2.3.1. FMS™ Test

The FMS [25] is a screening tool comprised of seven specific tests to assess an individual's overall functional movement capacity. Tests are scored on a 0–3 ordinal scale and include the squat, hurdle step, forward lunge, shoulder mobility, active straight leg raise (ASLR), push-up, and rotary stability. A score of 3 indicates that the subject was able to perform the movement correctly and without pain. A score of 2 indicates that the subject could complete the movement without pain but with some level of compensation. A score of 1 is given when the subject is unable to complete the movement as instructed. A score of 0 is recorded if the subject experiences pain with any portion of the movement. The overall FMS scores can range from 0 to 21. Kyle et al. pointed out in the FMS test that professional football players with lower scores on functional action screening are more likely to become injured than players with higher scores.

#### 2.3.2. Vertical-Jump Test and Dynamic Balancing Test

In the vertical jump test (Vertical Jump Test, VJ), tester uses the Opto Gait Italian intelligent motion analysis system (Optojump, Microgate, Bolzano, Italy) [26]. The test starts with the setting of the Vertec in which the standing height of the participant with one arm fully extended upward is taken to set the lowest pane. Participants are required to jump up and touch the highest possible pane. Participants were allowed to swing their arms and bend their knees to simulate the real movement in sports, tested 3 times, and take the highest test record score among them. The dynamic balance test uses the single-leg side hop test, and athletes should remain standing on one foot and jump to the opposite side with full on the ground with the other foot. When landing on one foot, they keep their body above 3 s to record effective results.

#### 2.3.3. Strength Test

Using Brown, Fleck and Kraemer [27, 28] study the maximum intensity prediction formula:

1RM = (number of repetitions × 0.0338 + 0.9849)× repetition weight. This index test needs to be measured by squatting. Squatting requires that the thighs are parallel to the ground, the projection point of the knee joint is controlled within the toes, and the torso remains upright.

#### 2.3.4. Sprint Test and Agility Test

The speed test 30 m sprint test (ST) uses a Brower Timing Systems speed tester (Draper, UT, USA) [29]. The subjects used a standing start at the designated starting line and started running on their own when they were ready. The best result was recorded from 2 tests. The accuracy of the test time was 0.01 s. Before the formal test, the participants were required to warm up for 15 minutes, wear a heart rate monitor, and only start the second test when the real-time heart rate recovery was lower than 100 bmp. The Arrowhead Agility Test (AAT) [30] mainly tests the athlete's ability to accelerate, decelerate, and change direction while running.

#### 2.3.5. Low Center of Gravity Run [31]

**Figure 1:**
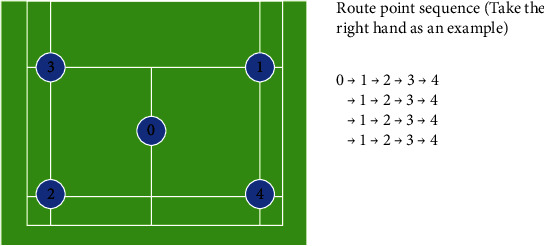
Diagram of running with a low center of gravity.

The test was designed to measure both repeated sprint and COD abilities. The LCGR test consisted of 20 repetitions of a pattern, with each repetition. The basic pattern is defined as the following: from the center of the court, the player moves around the 4 corners for repetitions (for a total of 20 movements) in the order of the number 1 (right forecourt), number 2 (left backcourt), number 3 (left forecourt), and number 4 (right backcourt), with a return to the center court number 0. Running to 1 corner and then returning to the center court, running to 2 corner and then running to 3 corner, returning to the center court, running to 4 corner and then running to 1 corner, returning to the center court. The players were instructed to touch each singles sideline with the hand to return the next one immediately (Figure 1). Running to 1 corner and then returning to the center court was considered as 1 movement.

#### 2.3.6. Double Swing Rope Skipping

Using a skipping rope counter, the test team members are required to swing the rope with both hands, jump once, and pass the rope twice under their feet. During the test, if there is an interruption caused by subjective reasons, the watch will not stop until 1500 skips are completed, and the completion time (s) is recorded.

#### 2.3.7. 1 Minute Batting against Wall

The tester stands about 2 m away from the wall, holding a low hand position to catch the ball. When the center of gravity moves forward, the nonholding arm raises its natural posture to maintain balance and strikes the ball continuously for 1 minute to the specified hitting area of the wall. If the fault causes the interruption, it is necessary to restart the timing and count the number of hits.

## 3. Statistical Analyses

All data are displayed as the mean and standard deviation and were initially computed as the mean values and SD in Microsoft Excel, and all additional analyses were computed in SPSS21.0. All data were checked for normality using the S-W test. One-way ANOVA was used between the groups, and a paired sample *t*-test was used to test the results before and after the test. A statistically significant difference was defined as *P* < 0.05, and a very significant difference was defined as *P* < 0.01. Cohen's *d* value is used to judge the effect (effect size) of the data measured before and after the group within the group. The effect size evaluation standard was divided into a large effect (ES > 0.8), a medium effect (0.5 < ES < 0.8), and a small effect (0.2 < ES < 0.5) [32].

## 4. Results

No injuries or overuse syndromes occurred during testing or training. The attendance rate of the two groups of participants in the training process was 96.8% (high-risk group) and 93.7% (low-risk group) separately, which made the experimental data convincing.

### 4.1. FMS Test Results

All data were normally distributed (*P* > 0.05), performance of motor functions, as compared with the pretest (ES = −0.81), the posttest of the high-risk group (HG) of the FMS total score had a very significant increase (*P* < 0.01). Compared with the pretest (ES = 0.65), the posttest of the low-risk group (LG) was significantly improved (*P* < 0.05). In the subtest of motor function, the posttest hurdle step score (ES = 0.61), active straight knee lifts score (ES = 0.60), and rotation stability score (ES = 0.65) of the high-risk group compared with the pretest scores had very significant improvements (*P* < 0.01). The deep squat score (ES = 0.60), shoulder flexibility score (ES = −0.58), and trunk stability push-up score (ES = −0.58) were significantly improved compared with the pretest scores (*P* < 0.05). Compared with the previous test, the low-risk group's posttest hurdle step score (ES = 0.55), shoulder flexibility score (ES = 0.16), active straight knee lift score (ES = 0.45), and rotation stability score (ES = −0.72) showed significant improvements. Except for the significant difference in the total FMS score between the high-risk group and the low-risk group in the posttest (*P* < 0.05), the results of the other subtests did not change significantly (Table 5).

**Figure 2:**
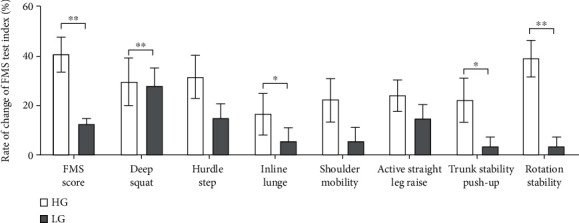
The index change rate before and after training.

After 8 weeks of INT intervention, there were significant differences in the FMS subindices between the low-risk group and the high-risk group, as well as in the change rates of the two groups before and after the experimental intervention (Figure 2).

There was a significant difference in the rate of change of the two parameters of hurdles and trunk stability pushups (*P* < 0.05); the rate of change of the FMS total score, deep squat, and rotation stability showed very significant differences (*P* < 0.01). Also, there was no significant difference in the rate of change of the three indexes of the in-line lunge, shoulder flexibility, and active straight leg raise.

### 4.2. Basic Athletic Ability Test Results

Basic athletic performance, the posttest vertical jump height (ES = 0.51), and dynamic balance value (left ES = 0.28 and right ES = 0.49) of the HG were significantly improved compared with the previous test (*P* < 0.01). There were significant differences in the posttest strength performance value (ES = 0.52), speed performance (ES = 0.23), and agility test (ES = 0.28) of the HG compared with the previous test (*P* < 0.05). There was no significant change in the test results of the other indicators (Table 6).

The comparison between the test results showed that there is a very significant difference between the high-risk group and the low-risk group in the sensitivity posttest group (*P* < 0.01), and there were significant difference between the dynamic balance ability, strength growth, and speed test performance posttest group (*P* < 0.05), but there was no significant difference between the test groups after the other tests.

### 4.3. Special Athletic Ability Test Results

In terms of specific athletic performance, the high-risk group's posttest low center of gravity run (ES = 0.76), double swing rope skipping (ES = 0.81), and a batting test against a wall (ES = 0.75) compared with the pretest showed a significant improvement (*P* < 0.01). The low-risk group's posttest low center of gravity run (ES = 0.74), double swing rope skipping (ES = 0.76), and batting test against a wall (ES = −0.63) were significantly different from the pre-test (*P* < 0.01).

The comparison of the results of the special athletic performance test between groups showed that there were very significant differences between the groups in the low center of gravity run and double swing rope skipping (*P* < 0.01), but no significant difference between the batting test against a wall (Table 7).

## 5. Discussion

Integrated neuromuscular training (INT) can improve athletes' motor function, performance, and efficiency. After 8 weeks of a training intervention in the high and low-risk groups, the scores for vertical jump, balance ability, strength, and batting test against a wall were significantly higher than before the intervention; the values of functional asymmetry, the speed test, and the agility test were significantly decreased, which was consistent with the research hypothesis. The results of this study are consistent with the conclusions of other related studies [21, 23, 24, 28, 33] and Schneiders et al. [34]. Previous research showed that soccer players with an FMS score of fewer than 14 points are 6 times more likely to be injured than other players. Tabatabaei et al. [35] found that compared with conventional training, neuromuscular training improves basketball players' FMS test scores and lower limb balance. Siberian [22] showed that over 8 weeks of Swiss Ball training intervention, 29 young badminton players underwent FMS tests and lower limb and upper limb balance tests in the 4th and 8th weeks, and their performance was significantly improved. Johnson et al. [36] showed that neuromuscular training has a good effect on improving the depth perception of the body and enhancing the sense of balance, focusing on strengthening the sacroiliac muscles to improve the athletes' dynamic balance ability. Combined with the results of previous studies, it has been found that Plyometric Training, core training, balance training, and resistance training in a program integrated neuromuscular training can change athlete's movement proficiency while reducing the risk of sports injuries.

### 5.1. The Impact of INT on Sports Injury Prevention

Injury is an inevitable part of the exercise process [37]. The cause of acute injury during exercise has become a focus of attention in sports training and sports human science [38]. Although badminton is not like football, rugby, and basketball, which has a direct physical confrontation, noncontact anterior cruciate ligament (ACL) injury is also very common. Research indicates that neuromuscular and biomechanical risk factors can be modified by the INT program design [24]. There are gender differences in the sensitivity of injury mechanism factors. Epidemiological studies on male and female athletes have found that the incidence of sports injuries in competitions is 2-5 times that in training, and the injury probability of female athletes is approximately 2-8 times higher than that of male athletes [2, 9, 22, 39]. Myer et al. and Van der Sluis et al. [40, 41] have shown that there is a certain correlation between INT reducing the incidence of joint injuries in athletes and age. The focus of training content is different at different ages, adolescence (13-17 years old) is mainly based on musculoskeletal development, without neuromuscular activation and adaptation, which also leads to frequent injuries caused by abnormal joint forces during exercise. Hewett et al. [42] found that neuromuscular training aimed at coordinated control of the body posture can successfully reduce the ACL injury rate by 60%. The left-right imbalance of the shoulder and trunk muscles has become an important factor in the high incidence of injuries after landing from a jump [23]. In training, badminton players should pay attention to strengthening the exercises on the nondominant side and improve the balance ability of badminton players through neuromuscular training, thereby reducing the incidence of sports injuries. Harati and Daneshmandi and Sedaghati et al. [43, 44] showed that core stability training can improve balance in swimmers helps with effective energy transfer and more coordinated actions.

Compared with athletes with better one-sided basic movement ability, excellent athletes have significantly higher completed movement quality and a lower injury incidence [45]. From a physiological point of view, the human body's balance ability is the concentrated expression of the human body's multisensory integration and movement control. The body mainly receives and inputs information through hearing, vision, and proprioception, information output [39]. The level of proprioception is one of the risk factors affecting neuromuscular damage [46]. However, INT combines the comprehensive effects of balance training, speed and sensitivity training, plyometric training, and coordination training, which can shorten proprioception and central nervous and motor system reaction times and improve injury resilience and motor performance abilities in athlete [46, 47]. Lu's [48] team conducted strength training on the lower limbs of the nondominant side of badminton players and found that these badminton players could reduce their asymmetry between the dominant side and the nondominant side through functional training, which significantly improved the strength and speed.

Movement functional training and auxiliary strength exercises can improve athletes' movement patterns and physical coordination [49]. Hewett et al.'s [50] research found that in the experimental group undergoing the INT intervention compared with the control group, the joint stability of female athletes in the experimental group was significantly improved (*P* < 0.05). Similar to the results of this study, the vertical jump performance of the experimental group was significantly improved (*P* < 0.05). The maintenance of joint stability is affected not only by a single tissue but also by a coordinated function of bones, joints, ligaments, muscles, tendons, and sensory receptor cortical nerve links [51]. Because female athletes show greater knee valgus angles when doing knee flexion support and exertion [52, 53], INT content-specific methods can be used to improve the sensorimotor system, increase neuromuscular control, and improve joint dynamic stability during strenuous exercise. Solomonow and Krogsgaard and Taimela et al. [54, 55] showed that the dynamic stability of joints depends on the multiple influences of ligaments, passive inhibition of joint mobility, and active neuromuscular control. A deficit in muscle strength will cause a response delay through the weakening of the spine's sense of position and neurological disorders [56].

### 5.2. The Effect of INT on Athlete Performance

The study of Chaouachi et al. [57] has shown that long-term use of a single training method can cause local tension of the muscles and nerves and reduce sensitivity to stimulation. Alternating training methods may help alleviate the overtraining effect caused by repeated high-load activities. Previous studies have shown that integrative neuromuscular training has a positive effect on balance ability [58–60], speed capability [10, 61, 62], muscle strength, muscle endurance, and flexibility [63] of the practitioner. Strength, balance, and body coordination contribute to the development of participant's motor skills and physical fitness, which is essential for building a healthier life. Ganeshkumar and Senthilkumar's research shows that the integrative neuromuscular training can effectively improve the speed ability, leg explosive power, and flexibility ability [64].

According to the concept of training specificity, a training event that includes targeted athletic ability can improve athletes' ability better than an event that does not have a specific targeted athletic ability [65]. Behm et al. [66] reviewed 18 studies involving balance training and concluded that the balance ability increased by an average of 105% in projects that included balance training goals. Balance ability can also be improved through strength training. The studies of Brooks et al. [67] and Sparkes and Behm [68] improve the ability and speed of proprioceptive muscles through strength exercises to change movement control, thereby improving the speed of the muscles and the ability to resist fatigue. When the limbs move asymmetrically, they will return to a more stable state after INT and improve the ability to jump vertically.

At the same time, Soligard et al. [69] studies have described that the resistance training effect of nonstable state and stable state in training has a certain correlation with the training experience of athletes, and the duration of a training intervention will affect the adaptability of the training effect. The INT program applied in our study aims to prevent the occurrence of sports injuries and neuroadaptability between the target muscles. The experimental results showed that the two groups of athletes have significant improvements in speed, agility, strength, and balance ability, but the actual intervention for the high-risk group had a better effect size than of the low-risk group. The results of Bonato et al. [70] on neuromuscular training to improve female nervous system control, stability, and proprioception and reduce the risk of lower back and lower limb injury in athletes showed that the test index of the intervention group was significantly higher than that of the control group, which was consistent with the results of this study. To study the influence of neuromuscular training on elite badminton players, Middleton et al. [71] conducted an RPT intervention for 8 weeks, and CMJ (+0.07 m), SLJ (+0.13 m), 1-MBT (+2.25 m), and 2-MBT (+0.26 m) were significantly increased, and the levels of CMJ, SLJ, 1-MBT, and 2-MBT were slightly decreased after 16 weeks of follow-up, while the speed was improved, which may be due to the influence of nontargeted physical training in the later period.

A study by Myer et al. [21] pointed out that a combination of high-intensity speed and strength training can effectively promote athletes' jumping and balance ability and improve the application efficiency of key technologies such as directional movement and rhythm control in competitions. Cormie et al. [72] found that after a period of intense strength training, the weight, fat-free mass, and BMI of the athletes remained unchanged. Because high-intensity strength training has a higher mechanical stimulation effect on the neuromuscular system, it increases the recruitment and emission frequency of the motor units, and after a long period (8 weeks or more) of integrated neuromuscular training, the athlete's 1RM strength has been improved by 12-33.2%. Macdonald et al.'s [73] research shows different strength training programs will have different results, but studies have confirmed that INT has a positive effect on improving power performance and is more effective than a single training method intervention. The increase in fatigue during exercise may be caused by a decrease in the excitability of muscle cell membranes caused by ion interference and excitation-contraction coupling damage [33]. In the research of Macquet and Fleurance [56], there are untimely shots, changes in field movements, and incorrect cognition, and the INT mode helps reduce the unstable performance of badminton players in harsh environments.

## 6. Limitations

This study has some limitations. A composite score of ≤14 on the FMS is commonly considered the threshold below which an individual is at potential risk of injury. However, the sensitivity of this method is only 24%, which indicates that 76% of athletes' pain will be ignored during the evaluation process [74]. The interpretation of FMS test results needs more research to verify. The research team was unable to obtain a complete record of the athlete's injury, which may be a factor that affects the result analysis. This study takes the female badminton players of the TJ Provincial Team as the research object. The small number of experimental objects will limit the application of statistical methods. Secondly, the representativeness of the data of female players in a team remains should be discussed. The impact of the INT method on athletes' long-term performance and injury prevention is not yet fully understood and needs further research.

## 7. Conclusion

Training methods that affect athletes' training performance and reduce the risk of sports injuries have long been the focus of global sports science. This paper uses integrative neuromuscular training to intervene female badminton players in different injury risk groups. The values of the monitoring indicators have been improved, but the effects of the changes are different. Furthermore, it shows that integrative neuromuscular training can improve balance (trunk and extremity), coordination, sensitivity, and exercise capabilities such as leg explosive power and speed capability. INT can help with physical preparation to enhance performance but also help reduce the risk of injuries. Screening tools such as the FMS can further identify deficiencies and links between INT and injury. This can help athletes maintain a longer competitive state in the international arena. In future research, the training stimulus produced by the minimum amount of training and its interaction with the training intensity which will maintain the athlete's exercise ability, which is an interesting content.

## Figures and Tables

**Table 1:** Functional movement screen results for female badminton players (*N* = 38).

	High-risk group (HG)	Low-risk group (LG)
Number	22	16
Test scores	10.24 ± 1.51	15.15 ± 1.41

**Table 2:** Anthropometric characteristics of participants (*N* = 38).

Group	Age	Height/m	Body mass/kg	BMI	Training years
HG (*N* = 22)	17 ± 1.8	1.70 ± 0.03	58.0 ± 2.4	19.23 ± 0.5	9 ± 1.3
LG (*N* = 16)	18 ± 1.1	1.72 ± 0.04	59.0 ± 3.1	19.66 ± 0.7	10 ± 1.3

**Table 3:** Integrative neuromuscular training program contents.

Objective	Practice content	Exercise load	Frequency time	Group count
Balanced and coordination capacity	BOSU ball lifts medicine ball on one leg twist	2 kg	20 s	2
	Bosu globular bridge lift	Bodyweight	30 s	2
	Balance board stands on one foot to catch the ball	Tennis	30 s	2
	Balance board stand with eyes closed on one foot	Bodyweight	30 s	2
	Swiss ball elbow plank	Bodyweight	60 s	2
	Swiss ball side plank	Bodyweight	60 s	2
	Swiss ball kneel catch	Tennis	30 s	2

Plyometric training	Reverse jump bar	Bodyweight	20	2
	Drop-jump practice	Bodyweight	15	2
	Jumping box practice	Bodyweight	20	2
	Fall standing long jump	Bodyweight	10	2
	Throw the ball with both hands on one side	2 kg	10	3
	Throw the ball forward with both hands	2 kg	10	3
	Turning and throwing medicine ball	2 kg	8	3

Speed and sensitivity	Hexagon jump	Bodyweight	30 s	2
	Agility ball exercise	Agileball	20	2
	Reverse sprint on your back	10 m	2	2
	Illinois agility training	Bodyweight	2	2
	Lie on back heel push	Bodyweight	20	2

Core stability	Elastic hip bridge	30P	40 s	2
	Lie on your back and twist	Bodyweight	30 s	2
	Overhanging medicine ball to lift knee	2 kg	15	2
	Hip bridge support on one leg	Bodyweight	30 s	2
	Kneel to support the limb on the other side	Bodyweight	20	2
	Reverse abdominal curl and knee lift on stool	Bodyweight	20	2
	Medicine ball body tilted forward	2 kg	10	2

Resistance training	Burpee	Bodyweight	15	2
	The climbing steps	Bodyweight	50 s	2
	Jumping jacks	Bodyweight	50 s	2
	Hundred push-ups	Bodyweight	20	2
	Russian abdominal rotation exercise		50 s	2

**Table 4:** Arrangement of integrated neuromuscular training content.

	Monday (a.m.)	Tuesday (a.m.)	Thursday (a.m.)	Friday (a.m.)
Training content	Balanced and coordination capacity	Speed and sensitivity	Balanced and coordination capacity	Speed and sensitivity
	Plyometric training core stability	Resistance training	Plyometric training core stability	Resistance training

**Table 5:** FMS total score and test results (*N* = 38).

Test index	Statistic	Pre	Post
	Group		
FMS score	HG (*N* = 22)	10.67 ± 1.80	14.67 ± 0.87^∗∗#^
			ES = −0.81
	LG (*N* = 16)	14.33 ± 0.50	15.67 ± 1.0^∗∗^
			ES = 0.65

Deep squat	HG (*N* = 22)	1.33 ± 0.5	2.0 ± 0.5^∗^
			ES = 0.60
	LG (*N* = 16)	2.1 ± 0.01	2.33 ± 0.5
			ES = 0.3

Hurdle step	HG (*N* = 22)	1.56 ± 0.53	2.33 ± 0.51^∗∗^
			ES = 0.61
	LG (*N* = 16)	2.11 ± 0.33	2.67 ± 0.5^∗^
			ES = 0.55

Inline lunge	HG (*N* = 22)	1.67 ± 0.52	2.01 ± 0.01
			ES = 0.42
	LG (*N* = 16)	2.11 ± 0.33	2.01 ± 0.01
			ES = 0.21

Shoulder mobility	HG (*N* = 22)	1.56 ± 0.53	2.1 ± 0.01^∗^
			ES = −0.58
	LG (*N* = 16)	2.3 ± 0.21	2.01 ± 1.21^∗^
			ES = 0.16

Active straight leg raise	HG (*N* = 22)	1.89 ± 0.33	2.56 ± 0.53^∗∗^
			ES = −0.60
	LG (*N* = 16)	2.11 ± 0.34	2.56 ± 0.53^∗^
			ES = 0.45

Trunk stability	HG (*N* = 22)	1.56 ± 0.53	2.1 ± 0.01^∗^

Push-up			ES = −0.58
	LG (*N* = 16)	2.0 ± 0.01	2.1 ± 0.34
			ES = −0.20

Rotation stability	HG (*N* = 22)	1.11 ± 0.33	1.78 ± 0.44^∗∗^
			ES = −0.65
	LG (*N* = 16)	2.12 ± 0.01	2.86 ± 0.5^∗^
			ES = −0.72

HG: high-risk group; LG: low-risk group; Pre: pretesting; Post: posttesting; ES represents the magnitude of the effect compared to Post and Pre; ^∗^significant difference between pre- and posttest of the two groups, *P* < 0.05; ^∗∗^significant difference between the pre- and posttest of the two groups, *p* < 0.01. ^#^The significant with-group difference, *P* < 0.05.

**Table 6:** Physical fitness test results.

Index	HG		LG	
	Pre	Post	Pre	Post
Vertical jump (cm)	33.69 ± 3.12	37.21 ± 2.83^∗∗^ ES = 0.51	35.76 ± 2.28	38.46 ± 2.08^∗∗^ ES = 0.53

Dynamic balance (L)	123.56 ± 5.57	126.78 ± 5.38^∗∗#^ ES = 0.28	127.33 ± 8.46	131.0 ± 6.18^∗^ ES = 0.24

Dynamic balance (R)	124.11 ± 5.68	129.11 ± 2.87^∗∗#^ ES = 0.49	127.56 ± 6.50	130.89 ± 6.07^∗^ ES = 0.26

Strength test	52.11 ± 2.80	56.22 ± 3.87^∗^ ES = 0.52	54.56 ± 1.04	57.11 ± 3.33 ES = 0.46

Sprint test	4.26 ± 0.13	4.21 ± 0.08^∗^ ES = 0.23	4.23 ± 0.14	4.12 ± 0.13^∗^ ES = 0.38

Agility test	17.39 ± 0.57	17.04 ± 0.63^∗##^ ES = 0.28	17.07 ± 0.62	16.8 ± 0.60^∗∗^ ES = 0.21

L: left; R: right. ^∗^Significant difference between the pre- and posttest of the two groups, *P* < 0.05; ^∗∗^very significant difference between pre- and posttest of the two groups, *P* < 0.01. ^#^The significant with-group difference, *P* < 0.05; ^##^very significant with-group difference, *P* < 0.01.

**Table 7:** Special athletic ability test results.

Index	HG		LG	
	Pre	Post	Pre	Post
Low center of gravity run (sec)	38.22 ± 1.23	35.68 ± 0.94^∗∗##^ ES = 0.76	37.02 ± 1.40	34.35 ± 1.03^∗∗^ ES = 0.74

Double swing rope skipping (sec)	23.02 ± 1.84	19.19 ± 0.54^∗∗##^ ES = 0.81	21.08 ± 1.60	17.65 ± 1.30^∗∗^ ES = 0.76

Batting test against a wall	112.89 ± 8.36	130.89 ± 7.42^∗∗^ ES = −0.75	114.11 ± 11.24	132.33 ± 10.94^∗∗^ ES = −0.63

^∗^Significant difference between the pre- and posttest of the two groups, *P* < 0.05; ^∗∗^very significant difference between pre- and posttest of the two groups, *P* < 0.01. ^#^The significant with-group difference, *P* < 0.05; ^##^very significant with-group difference, *P* < 0.01.

## Data Availability

The data involved in the paper has been derived from the original data analysis of the experiment.
